# Autoimmune Switch From Graves’ Disease to Hashimoto’s Thyroiditis With Coexisting Sjogren’s Syndrome: A Case of Polyautoimmunity in a Young Woman

**DOI:** 10.7759/cureus.59446

**Published:** 2024-05-01

**Authors:** Shalini Sri Kumaran

**Affiliations:** 1 Internal Medicine, Singapore General Hospital, Singapore, SGP

**Keywords:** sjogren’s syndrome, autoimmune disease, distal renal tubular acidosis, thyroid antibodies, polyautoimmunity, autoimmune thyroid disorders

## Abstract

Autoimmune thyroid disease (AITD) encompasses a spectrum of conditions ranging from Graves’ disease (GD) to Hashimoto’s thyroiditis (HT). These conditions often coexist with other autoimmune diseases (AIDs). This case describes a young woman in her 20s who transitioned from GD to HT during her first pregnancy, while having another coexisting AID, Sjogren’s syndrome (SS). AITD and SS are recognized as the most common polyautoimmune diseases, sharing many common pathophysiological characteristics such as the presence of lymphocytic infiltrates, similar expressions of the human leukocyte antigen molecules, and predisposing environmental factors. This case underscores the importance for physicians to be vigilant regarding the possibility of changing antibodies in AITD and the potential for concurrent AIDs in a single individual. It highlights the need for screening such patients for comprehensive management.

## Introduction

Autoimmunity occurs when there is a breakdown of tolerance to self-antigens and conditions caused by such reactions are known as autoimmune diseases (AIDs) [1,2]. AIDs can be classified as organ-specific, such as autoimmune thyroid disease (AITD), or systemic diseases, such as rheumatoid arthritis [1,3,4].

Polyautoimmunity, the coexistence of two or more AIDs in an individual [2-4], was first described by Sheenan and Stanton-King [3]. It refers to the presence of multiple well-defined AIDs in a single person and has an estimated global prevalence of 0.5% [4].

The development of AID involves complex mechanisms, including interactions between genetic, epigenetic, and environmental factors, known as autoimmune tautology [3,4].

This case report describes a young woman with polyautoimmunity who was initially diagnosed with Graves’ disease (GD) and later transitioned to Hashimoto’s thyroiditis (HT) during her pregnancy, all the while possibly having another coexisting AID, Sjogren’s syndrome (SS).

## Case presentation

A 25-year-old female presented to the hospital at 11 weeks gestation with symptoms of lower limb weakness and difficulty walking after dinner that night. She had a known history of GD since the age of 20 but had been non-compliant with her medication for two years at presentation. On examination, she was alert and coherent. Vital signs were as follows: temperature (T) of 36.9°C, blood pressure (BP) of 128/69 mm/Hg, pulse of 121 beats per minute (bpm) with a regular rhythm, and oxygen saturation (SpO_2_) of 99% on ambient room air. Mild proptosis was noted, but there was no evidence of goiter or hand tremors. Neurological examination revealed normal cranial nerves and normal power in bilateral upper limbs. However, there was a proximal lower limb weakness pattern, with power rated as 3/5 and 4/5, respectively. Tone, reflexes, and sensation in both upper and lower limbs were preserved. Cardiovascular, respiratory, and abdominal examination was unremarkable.

Initial investigations (Table 1) revealed hypokalemia and a hyperchloremic metabolic acidosis with the following results: potassium (K^+^) of 2.0 mmol/L, sodium (Na) of 135 mmol/L, magnesium (Mg^2+^) of 0.87 mmol/L, chloride (Cl^-^) of 110 mmol/L, and bicarbonate of (HCO_3^-^_) 12.7 mmol/L. Additionally, her thyroid panel was consistent with GD, showing suppressed thyroid-stimulating hormone (TSH) of <0.01 mIU/L, elevated free thyroxine 4 (fT4) of 26.3 pmol/L, and fT3 of 7.3 pmol/L, with a positive TSH receptor antibody (TRAb) 4.85 IU/L. She was diagnosed with hypokalemic periodic paralysis secondary to poorly controlled GD, along with a newly identified normal anion-gap metabolic acidosis suspected to be caused by renal tubular acidosis (RTA), which required further investigation. Under the supervision of the endocrinology team, she was initiated on propylthiouracil (PTU) 100 mg twice daily and intravenous (IV) potassium chloride (KCl) replacement during her hospitalization. The patient’s proximal myopathy resolved upon normalization of her K^+^ levels, and she was discharged well on PTU and oral potassium citrate replacement. Plans were made for her to follow up in the joint obstetric-medical clinic in four weeks.

**Table 1:** Kidney and thyroid panel results from the first pregnancy. TSH = thyroid-stimulating hormone; TRAb = TSH receptor antibody

Test	Reference range	Patient’s results
Potassium (K^+^)	3.5–5.0 mmol/L	2.0 mmol/L
Sodium (Na)	136–146 mmol/L	135 mmol/L
Magnesium (Mg^2+^)	0.74–0.97 mmol/L	0.87 mmol/L
Chloride (Cl^-^)	100–107 mmol/L	110 mmol/L
Bicarbonate (HCO_3-_)	19.0–29.0 mmol/L	12.7 mmol/L
TSH	0.65–3.70 mIU/L	<0.01 mIU/L
Free T4	8.8–14.4 pmol/L	26.3 pmol/L
Free T3	3.2–5.3 pmol/L	7.3 pmol/L
TRAb	<1.76 IU/L	4.85 IU/L

Unfortunately, the patient attended her follow-up appointments only in the later stages of her pregnancy, despite multiple reminders and calls. A thyroid panel repeated at 27 weeks gestation showed a decreasing fT4, leading to a reduction in her PTU dosage. Subsequently, PTU was discontinued at 33 weeks gestation when her fT4 levels dropped to the hypothyroid range, as shown in Table 2. Unfortunately, a TRAb level was not repeated at this juncture. At 37 weeks gestation, due to fetal complications, she underwent an emergency cesarean section and delivered a healthy baby boy. At this juncture, she was biochemically hypothyroid, with TSH of 5.45 mIU/L and fT4 of 8.4 pmol/L, although she remained clinically asymptomatic. Post-delivery, she was planned to be seen in the outpatient setting within four weeks to repeat her thyroid panel and further investigate the new metabolic acidosis.

**Table 2:** Thyroid panel trend in the first pregnancy. TSH = thyroid-stimulating hormone; N/A = not assessed

Test	Reference range	11 weeks gestation	27 weeks gestation	33 weeks gestation	37 weeks gestation
TSH	0.65–3.70 mIU/L	<0.01 mIU/L	<0.01 mIU/L	1.74 mIU/L	5.45 mIU/L
Free T4	8.8–14.4 pmol/L	26.3 pmol/L	10.4 pmol/L	7.4 pmol/L	8.5 pmol/L
Free T3	3.2–5.3 pmol/L	7.3 pmol/L	N/A	3.7 pmol/L	N/A

Regrettably, the patient defaulted on all her postpartum appointments and only returned a year later during her second pregnancy at 19 weeks gestation. Clinically, she still had mild proptosis but was otherwise euthyroid. Her vital signs were as follows: temperature of 36.4°C, BP of 112/86 mm/Hg, regular pulse rate of 86 bpm, and SpO_2_ of 100% on room air. Neurologically, she presented with a similar proximal myopathy pattern weakness in bilateral lower limbs with power in the proximal and distal muscles being 3+/5 and 4+/5, respectively. The rest of her neurological, cardiovascular, respiratory, and abdominal examination was unremarkable except for a gravid uterus.

The repeat kidney panel once again revealed findings consistent with hypokalemia and hyperchloremic metabolic acidosis: K^+^ of 2.3 mmol/L, Na of 137, Mg^2+^ of 0.92 mmol/L, Cl^-^ of 110 mmol/L, and HCO_3^-^_ of 14 mmol/L, and her thyroid panel was indicative of hypothyroidism, with TSH of 11.9 mIU/L and fT4 of 8.0 pmol/L (Table 3). Her TRAb levels were undetectable this time, and she had an elevated thyroid peroxidase antibody (TPOAb) level of 2166 IU/mL.

**Table 3:** Kidney and thyroid panel results from the second pregnancy. TSH = thyroid-stimulating hormone; TRAb = TSH receptor antibody; TPOAb = thyroid peroxidase antibody

Test	Reference range	Patient’s results
Potassium (K^+^)	3.5–5.0 mmol/L	2.3 mmol/L
Sodium (Na)	136–146 mmol/L	137 mmol/L
Magnesium (Mg^2+^)	0.74–0.97 mmol/L	0.92 mmol/L
Chloride (Cl^-^)	100–107 mmol/L	110 mmol/L
Bicarbonate (HCO_3-_)	19.0–29.0 mmol/L	14.0 mmol/L
TSH	0.65–3.70 mIU/L	11.9 mIU/L
Free T4	8.8–14.4 pmol/L	8.0 pmol/L
TRAb	<1.76 IU/L	<1.10 IU/L
TPOAb	<9.0 IU/mL	2166 IU/mL

She was diagnosed with HT and commenced on levothyroxine 50 µg daily. Further investigations for RTA yielded the following results: the presence of antinuclear antibody (ANA) titer-1: 1:>640 with a speckled pattern, ANA titre-2: 1:>640 with a nucleolar pattern, positive anti-extractable antigen (ENA) screen, absence of anti-double stranded DNA (anti-dsDNA), positive rheumatoid factor (RF), elevated urine protein/creatinine ratio of 1.1g/dL, and a urine pH of 8.0 (Table 4), raising the possibility of another AID, likely SS. At 34 weeks gestation, her anti-Ro and anti-La results were pending. Her most recent thyroid panel showed TSH of 4.45 mIU/L and fT4 of 11.1 pmol/L. She is being managed by a multidisciplinary team involving obstetricians, rheumatologists, obstetric medicine specialists, and neonatologists.

**Table 4:** Renal tubular acidosis investigation panel. ANA = antinuclear antibody; Anti-dsDNA = anti-double-stranded DNA; ENA = extractable nuclear antigen; RF = rheumatoid factor; UPCR = urine protein/creatinine ratio

Test	Reference range	Patient’s results
ANA titer-1	1:160: borderline, 1:320: weak positive, 1:640 or higher: positive	1:>640 speckled pattern
ANA titer-2	1:160: borderline, 1:320: weak positive, 1:640 or higher: positive	1:>640 nucleolar pattern
Anti-dsDNA	<25 IU: negative, 25–30 IU: indeterminate, >30 IU: positive	5.17 IU
ENA screen	Positive or negative	Positive
RF	<14.0 U/mL	18.4 U/mL
UPCR	≤0.2 g/L	1.1 g/L
pH, urine	4.8–7.4	8.0

## Discussion

AITD is the most common organ-specific AID [1-3,5] and represents a spectrum of conditions, with GD and HT lying on opposite ends, encompassing other disorders such as subacute thyroiditis, postpartum thyroiditis, and Graves’ orbitopathy [1,6]. Phenotypically, GD presents with hyperthyroid symptoms, whereas HT presents with hypothyroid symptoms. However, there is an overlap in the antigens implicated in these conditions, which may fluctuate at times [1,6,7].

Similarly, SS manifests as a spectrum of diseases, ranging from xerostomia and keratoconjunctivitis (sicca symptoms) to more organ-specific or systemic involvement, affecting systems such as the musculoskeletal, pulmonary, gastrointestinal, hematological, renal, and neurological systems [5].

AITD and SS are recognized as the most frequently coexisting AIDs in a single patient [3,5], with the prevalence of SS in AITD ranging from 3% to 32% and AITD in SS from 10% to 30%, although these figures vary due to study heterogeneity [7]. These patients are also at a heightened risk of developing other AIDs [2,7].

The cascade of inflammatory glandular insult observed in SS mirrors the pathophysiology of AITD [8]. Common predisposing factors include female predominance, prior viral infections (Epstein-Barr virus, herpes simplex virus), and shared genetic backgrounds, such as the expression of human leukocyte antigen (HLA) molecule class II: HLA-DR3 and HLA-B8 [5,7,8].

In both conditions, autoantibodies play a crucial role, leading to T and B-cell lymphocytic infiltration in the exocrine and thyroid glands, respectively [1,5,7,8]. The main antibodies implicated in AITD include TPOAb, thyroglobulin antibody, and TRAbs [1]. TPOAb was one of the first antibodies to be discovered and considered the hallmark of HT, while TRAbs are characteristic of GD [1]. TRAbs can be classified as stimulating (TSAbs), blocking (TBAbs), or neutral antibodies.

TSAbs are initially produced in GD, but an individual’s immune state may fluctuate due to the intricate interplay of genetic and environmental factors, resulting in a switch to TBAbs or neutral TRAbs. This balance influences the disease phenotype and biochemical findings [1,9]. TRAb levels begin to decline at 20 weeks gestation due to gestational immune modulation, and thionamide therapy primarily reduces TRAb through its immunomodulatory effects [9]. Laboratories using receptor assays often do not differentiate between TSAbs and TBAbs, limiting the predictive accuracy of the disease phenotype [9]. The transition from GD to HT in our patient was likely due to these two factors, leading to a shift in the balance of TSAbs to TBAbs.

Similarly, autoantibodies frequently associated with SS include ANA, RF, and anti-ENA, with anti-Ro and anti-La being the most common anti-ENAs. [10]. A study by D’Arbonneau et al. [11] showed that the presence of RF and anti-Ro antibodies, in addition to thyroid antibodies, may indicate susceptibility to thyroid disorders.

Hypothyroidism is the most common clinical manifestation of AITD in SS, often preceding the diagnosis of SS [7,8]. Nonetheless, a recent study by Zeher et al. [12] suggested that AITD may manifest before or after the onset of SS. Renal involvement is a well-known extraglandular manifestation of SS, encompassing interstitial nephritis with proximal or distal RTA, tubular proteinuria, glomerular disease, or renal failure [13]. The pathogenesis of distal RTA involves immunological tubular assault, leading to hypokalemia [13,14].

The patient in this case lacked typical sicca symptoms but presented with more renal manifestations. Looking back, her initial presentation of hypokalemic paralysis and metabolic acidosis likely represented a renal manifestation of her undiagnosed SS.

## Conclusions

This case underscores the diverse presentation of AIDs and the phenomenon of polyautoimmunity. Our patient was initially diagnosed with GD in her early 20s and later developed SS, although pinpointing the exact onset is challenging due to the absence of common symptoms such as dry eyes and dry mouth. During her pregnancy, she transitioned from GD to HT, likely due to the immunomodulatory effects of both pregnancy and anti-thyroid therapy. However, this transition was not biochemically evident due to limitations in our laboratory testing.

Furthermore, this case prompts consideration of screening certain patients with AITD for SS and vice versa, given their frequent coexistence. Given cost-effectiveness, women of childbearing age may constitute a relevant subgroup for such screening, as AIDs are prevalent in this demographic, and pregnancy represents an immunomodulatory phase. Screening could involve conducting a comprehensive medical history and being attentive to the potential manifestations of other AIDs. If any of these symptoms are identified, further laboratory tests may be warranted. This screening approach could significantly impact immediate management strategies and prognosis, given the heightened risk of developing other autoimmune conditions.

Finally, this case also highlights the importance of heightened awareness among clinicians when caring for young women and the adoption of a multidisciplinary approach, especially considering that young women may only present during pregnancy.
